# Serum Diamine Oxidase as a Hemorrhagic Shock Biomarker in a Rabbit Model

**DOI:** 10.1371/journal.pone.0102285

**Published:** 2014-08-21

**Authors:** Liang Zhao, Lin Luo, Weikun Jia, Juan Xiao, Gang Huang, Geng Tian, Jingwei Li, Yingbin Xiao

**Affiliations:** Department of Cardiovascular Surgery, Xinqiao Hospital, Third Military Medical University, Chongqing, China; German Red Cross Blood Service Frankfurt, Germany

## Abstract

**Background:**

In prolonged hemorrhagic shock, reductions in intestinal mucosal blood perfusion lead to mucosal barrier damage and systemic inflammation. Gastrointestinal failure in critically ill patients has a poor prognosis, so early assessment of mucosal barrier injury in shock patients is clinically relevant. Unfortunately, there is no serum marker that can accurately assess intestinal ischemia-reperfusion injury.

**Objective:**

The aim of this study was to assess if serum diamine oxidase levels can reflect intestinal mucosal injury subsequent to prolonged hemorrhagic shock.

**Methods:**

Thirty New Zealand white rabbits were divided into three groups: a control group, a medium blood pressure (BP) group (exsanguinated to a shock BP of 50 to 41 mm Hg), and a low BP group (exsanguinated to a shock blood pressure of 40 to 31 mm Hg), in which the shock BP was sustained for 180 min prior to fluid resuscitation.

**Results:**

The severity of hemorrhagic shock in the low BP group was significantly greater than that of the medium BP group according to the post-resuscitation BP, serum tumor necrosis factor (TNF)-α, and arterial lactate. Intestinal damage was significantly more severe in the low BP group according to Chiu’s scoring, claudin-1, intercellular adhesion molecule (ICAM)-1, and myeloperoxidase expression. Serum diamine oxidase was significantly increased in the low BP group compared to the medium BP and control groups and was negatively correlated with shock BP.

**Conclusion:**

Serum diamine oxidase can be used as a serological marker in evaluating intestinal injury and shows promise as an indicator of hemorrhagic shock severity.

## Introduction

In the battlefield or remote areas, prolonged hemorrhagic shock often results in multiple organ failure (MOF) with a mortality rate of 24% [1]–[4]. The gut is considered to be the “motor” driving MOF [5]– because hemorrhagic shock reduces intestinal mucosal blood perfusion that leads to intestinal mucosal barrier damage, intestinal barrier dysfunction, ectopic flora, endotoxemia, systemic inflammation, and finally MOF [8]–[13]. Clinical studies have revealed that traumatic hemorrhagic shock patients with MOF display significantly higher levels of inflammatory cytokines (e.g., tumor necrosis factor (TNF)-α, interleukin (IL)-1, IL-6) than those without MOF [14], and animal experiments have shown that severe system inflammation is strongly related with prolonged hemorrhagic shock [15].

The timing of resuscitation and the severity of hemorrhagic shock are critical factors affecting intestinal damage. Leukocyte activation and resulting oxidative stress occurs three to six hours after traumatic hemorrhagic shock and is an important factor underlying mucosal barrier damage [16]–[19]. Activated leukocytes, which adhere to vascular endothelial cells via immunoglobulin and intercellular adhesion molecule (ICAM)-1, release peroxides that destroy the intestinal mucosal barrier [20]–[23]. As this gastrointestinal failure in critically ill patients has a poor prognosis [24]–[26], early assessment of mucosal barrier injury in shock patients is clinically relevant.

Unfortunately, there is no serological marker that can accurately assess intestinal ischemia-reperfusion injury. Some studies have found that serum diamine oxidase (DAO) levels are significantly increased after acute mesenteric ischemia [27]–[30], but it is unclear whether serum DAO levels can function as a serological marker in evaluating the severity of intestinal injury induced by hemorrhagic shock.

In this study, a hemorrhagic shock model was constructed with New Zealand rabbits in which two different levels of shock blood pressures (BPs) were maintained for 180 minutes in order to simulate two degrees of prolonged hemorrhagic shock. We hypothesized that prolonged hemorrhagic shock would cause obvious intestinal damage and that serum DAO can be used as an indicator to reflect the differing severities of intestinal ischemia-reperfusion injury accompanying the two degrees of prolonged hemorrhagic shock.

## Materials and Methods

### Animal Subjects

All the following procedures were strictly consistent with 3R principles, while animal care and housing procedures were in compliance with Chinese regulatory requirements. The protocol of this study was approved prior to implementation by the Ethics Committee of Third Military Medical University, and all procedures were in accordance with the National Institutes of Health Guidelines for Animal Research (Guide for the Care and Use of Laboratory Animals). Special care was taken to minimize number of and suffering of animals. Thirty male New Zealand white rabbits (weighing 2.4 to 2.6 kg, aged 6 to 8 months) were purchased from animal facility at Third Military Medical University (Chongqing, China). The rabbits were housed in individual cages and received humane care according to the Principles of Laboratory Animal Care. The laboratory temperature was maintained at 24±1°C.

### Experimental Protocol

The 30 rabbits were randomly divided into three groups based on their shock BP levels: the control group, the medium BP group (50 to 41 mm Hg), and the low BP group (40 to 31 mm Hg). All animals were fasted overnight and had free access to water before the experiment. Then, the rabbits were anesthetized by administration of 30 mg/kg 3% pentobarbital, and 20-gauge venous indwelling needles were inserted into the right and left femoral arteries to measure BP and to withdraw arterial blood samples, respectively. A central venous pressure catheter (7.5 Fr.; Baxter Healthcare, Deerfield, IL, USA) was positioned in the right atrium via the right jugular vein for the collection of venous blood samples. The animals were allowed to stabilize for 30 min before induction of hemorrhagic shock.

After a baseline BP was obtained, BP-controlled hemorrhagic shock was induced. The withdrawn blood was collected and heparinized with 10 units/ml heparin. BP was continually monitored and recorded every 2 min. We first used a 180-min prolonged hemorrhagic shock animal model based on the timing of neutrophil activation. The time points corresponding to the initiation of bleeding, the end of prolonged hemorrhagic shock, and 120 min after resuscitation were marked T1, T2, and T3, respectively. Because of autologous transfusion, the shock period from T1 to T2 was separated into two phases. During the first phase, blood was withdrawn at a fast rate of 1 ml/kg/min until the BP reached the target pressure. During the second phase, blood withdrawal or reperfusion was cautiously performed at a slow rate of 0.5 to 1 ml/unit time to stabilize the shock BP. Resuscitation began with equal volumes of heparinized blood and Ringer’s solution at T2 at a rate of 2 ml/kg/min. The experiment was concluded at T3 by euthanizing the rabbits through an overdosed intravenous injection of phenobarbital.

### Specimen Preparation

The 1-ml venous blood samples were drawn at T1, T2, and T3 with simultaneous infusion of an equal volume of Ringer’s solution. The blood samples were centrifuged at 3500 rpm for 15 min at 4°C. After waiting 30 minutes for coagulation, the supernatant was separated and stored at −80°C for subsequent measurement of DAO, lactate, and TNF-α. Then, a 6.0 to 7.0-cm segment of intestine was resected 10 cm away from the terminal ileum (ileocecal valve) and divided into two segments. One segment was fixed using formalin and embedded in paraffin; the other section was washed with cold saline, dried with blotting paper, and preserved at −80°C.

### Scoring Intestinal Mucosal Injury

The embedded segments of small intestine from all three groups were stained with hematoxylin-eosin. The severity of injury was evaluated by two independent, blinded pathologists using Chiu’s scoring method [31]. Briefly, mucosal damage was scored from 0 to 5 as follows: 0, normal mucosal villi; 1, development of sub-epithelial Gruenhagen space at the apex of the villus; 2, extension of the sub-epithelial space with moderate lifting of the epithelial layer from the lamina propria; 3, massive epithelial lifting down the sides of villi, possibly with a few denuded tips; 4, denuded villi with the lamina propria and dilated capillaries exposed, possibly with increased cellularity of the lamina propria; and 5, digestion and disintegration of the lamina propria, hemorrhage, and ulceration.

### Intestinal Claudin-1 and ICAM-1

The embedded intestine was cut into 6-µm sections using a cryostat (LEICA CM 1800, Leica Co., Germany). The sections were further fixed with 4% paraformaldehyde at room temperature for 10 min and washed with PBS. The samples were then immunohistochemically stained with goat polyclonal antibodies against rabbit claudin-1 or rabbit ICAM-1 (diluted 1∶50). The secondary antibody (rhodamine-labeled rabbit anti-goat IgG diluted 1∶200) was added and incubated at 37°C for 30 min followed by washes with PBS and incubation with Hoechst 33342 (2 µg/ml) at room temperature for 3 min. After washes with PBS, the specimen was sealed and observed using a laser confocal microscope. Fluorescence intensity was detected at an excitation wavelength of 543 nm for R-phycoerythrin and 405 nm for Hoechst. Five fields of view (1.6×10^4^ µm^2^ each) in the rabbit intestinal mucosa were evaluated for each condition. Fluorescence intensities of claudin-1 or ICAM-1 were estimated by Image Pro Plus software and expressed as an average proportion of positive areas per one field of view (1.6×10^4^ µm^2^).

### Intestinal Myeloperoxidase Activity

Myeloperoxidase (MPO) activity in homogenates of the whole intestine (proximal jejunum) was determined as previously described [32]: “The tissues were washed and homogenized using Tekmar tissue homogenizer (Brinkman, Cincinnati, OH) for 20 secs on ice in potassium phosphate buffer (pH, 7.4) containing 2 mM PMSF. The homogenate was centrifuged at 10,000 rpm for 30 mins at 4°C (39.2°F). The pellet (90% of the total myeloperoxidase [MPO] activity), was homogenized in 10 volumes of ice-cold 50 mM potassium phosphate buffer (pH, 6.0) containing 0.5% hexadecyltrimethyl-ammonium bromide and 10 mM EDTA. MPO activity was determined by using a modification of the method (22) in which the enzyme catalyzes the oxidation of 3,3′,5,5′-tetramethylbenzidine by hydrogen peroxide. An aliquot of standard recombinant human MPO (0–0.125 units) or samples homogenate was added to 0.5 mL MPO cocktail (80 mM potassium phosphate buffer; pH, 5.4; 0.5% (w/v) hexadecyltrimethyl-ammonium bromide; and 1.6 mM 3,3′,5,5′-tetramethylbenzidine). The mixtures of samples with the MPO cocktail were placed at 37°C (98.6°F), and the reaction was started by addition of 0.3 mM hydrogen peroxide. The samples were incubated for 3 mins, and the reaction was stopped with 1 mL of chilled acetate buffer. The intensity of the blue chromogen of the reaction mixture (1 mL) that possessed a wavelength maximum at 655 nm was read. The number of units of MPO in the tissue homogenate was calculated from the plot of the standard MPO units vs. absorption. The MPO activity was recorded as number of units per mg of protein. Protein determination in the final homogenate was estimated by bicinchoninic acid (Pierce Chemical, Rockford, IL) protein method using total bovine serum albumin as a standard”.

### Serum DAO, Serum TNF-α, and Arterial Lactate

The supernatants from the blood samples at T1, T2, and T3 were collected for measurements of DAO and TNF-α. Serum DAO activity and TNF-α concentrations were measured using enzyme-linked immunosorbent assay (ELISA) kits (CUSABIO; Wuhan, China) performed according to the manufacturer’s instructions. Each sample was run in duplicate and assessed by an automated ELISA reader at 436/450 nm wavelength. Arterial blood samples collected at T1, T2, and T3 in all groups were analyzed for blood lactate concentration using a portable blood gas analyzer (i-STAT; Abbott, Abbott Park, IL, USA).

### Statistical Analysis

Data were presented as means ± SDs (n = 10 rabbits/group). Significance was evaluated using one-way analysis of variance (ANOVA) (SPSS 21.0; SPSS Inc., Chicago, IL, USA) followed by Turkey’s test for comparisons among groups. Statistical significance was set at *P*<0.05. Correlations between different variables were assessed by Pearson’s or Kendall’s coefficient.

## Results

### Hemodynamic Changes

**Figure 1:**
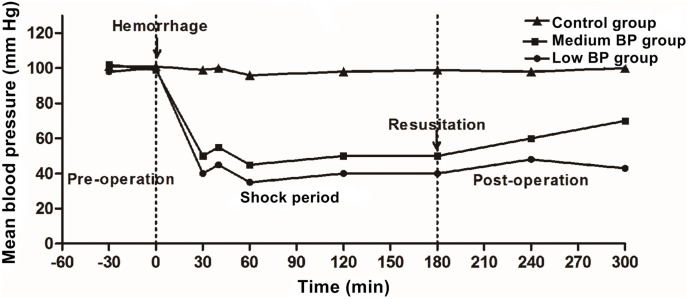
Time Course for the Prolonged Hemorrhagic Shock Model. Animals in the medium BP group or low BP group underwent hemorrhagic shock for 180 min at 41–50 mm Hg or 31–40 mm Hg, respectively, before fluid resuscitation. The control group was the sham shock group.

No animals died prior to sacrifice at T3. BP levels were not different at T1 (*P*>0.05). The procedure for prolonged shock-resuscitation is detailed in Figure 1. The BP in the control group was relatively stable, fluctuating from 108 mm Hg to 96 mm Hg. The mean arterial pressure (MAP) decreased over an approximate 30-min period to the target shock BP of 41 to 50 mm Hg in the medium BP group and 31 to 40 mm Hg in the low BP group. The MAP during the shock maintenance phase was 42.8±4.7 mm Hg in the medium BP group and 35.8±5.6 mm Hg in the low BP group. The shock maintenance phase was successfully prolonged to 180 min to delay the resuscitation after hemorrhagic shock. At T3, the MAP in the low BP group (41.7±5.1 mm Hg) was significantly lower than the MAP in the medium BP group (70.0±4.8 mm Hg, *P*<0.01).

### Intestinal Morphology

**Figure 2:**
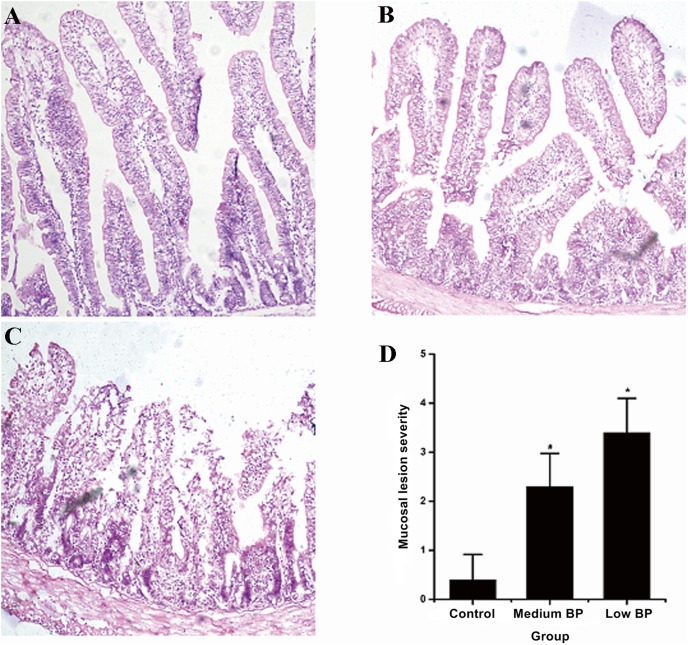
Prolonged Hemorrhagic Shock Causes Intestinal Mucosal Damage. A: control group; B: medium BP group; C: low BP group; and D: Chiu’s score quantifies intestinal mucosal damage. Animals in the medium BP group or low BP group underwent hemorrhagic shock for 180 min at 41–50 mm Hg or 31–40 mm Hg, respectively, before fluid resuscitation. The control group was the sham shock group. Data are shown as means ± SDs. *P<0.05 vs. control group or medium BP group, ^#^ *P*<0.05 vs. control group.

All animals were sacrificed at T3. The results of histological examination and Chiu’s scoring are shown in Figure 2. According to Chiu’s evaluating system, a higher Chiu’s score represents more severe intestinal damage. In the control group, the intestines were normal with a Chiu’s score of 0.40±0.52. In the medium BP group, the intestines manifested lesions with a Chiu’s score of 2.3±0.67. In the low BP group, the intestines exhibited more severe damage with a Chiu’s score of 3.5±0.53. The scores were significantly different between binary comparisons of the groups (*P*<0.01).

### Claudin-1 Expression

**Figure 3:**
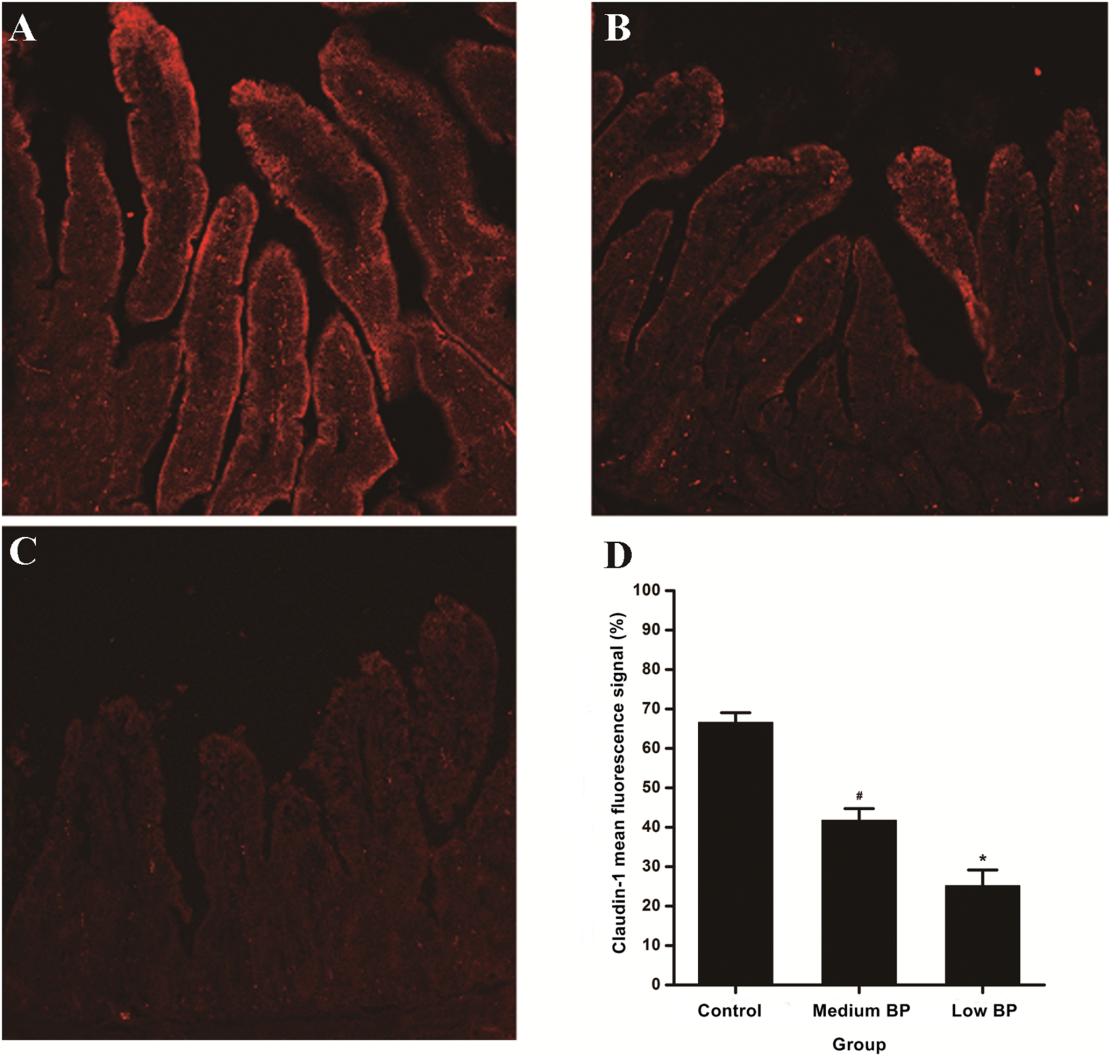
Intestinal Claudin-1 Expression. A: control group; B: medium BP group; C: low BP group; and D: mean fluorescence signal of claudin-1 (100%). Animals in the medium BP group or low BP group underwent hemorrhagic shock for 180 min at 41–50 mm Hg or 31–40 mm Hg, respectively, before fluid resuscitation. The control group was the sham shock group. Data are shown as means ± SDs. *P<0.05 vs. control group or medium BP group, ^#^ *P*<0.05 vs. control group.

As aberrant claudin-1 expression directly impairs mucosal barrier function, immunohistochemistry was used to detect claudin-1 expression in our prolonged hemorrhagic shock model (Fig. 3). While a normal fluorescence intensity was seen in cross-sections of the control group, claudin-1 expression at T3 in the low and medium BP groups showed significantly decreased fluorescence intensity (*P*<0.05), and the fluorescence intensity in the low BP group was significantly lower than that of the medium BP group (*P*<0.05).

### Intestinal ICAM-1 Expression

**Figure 4:**
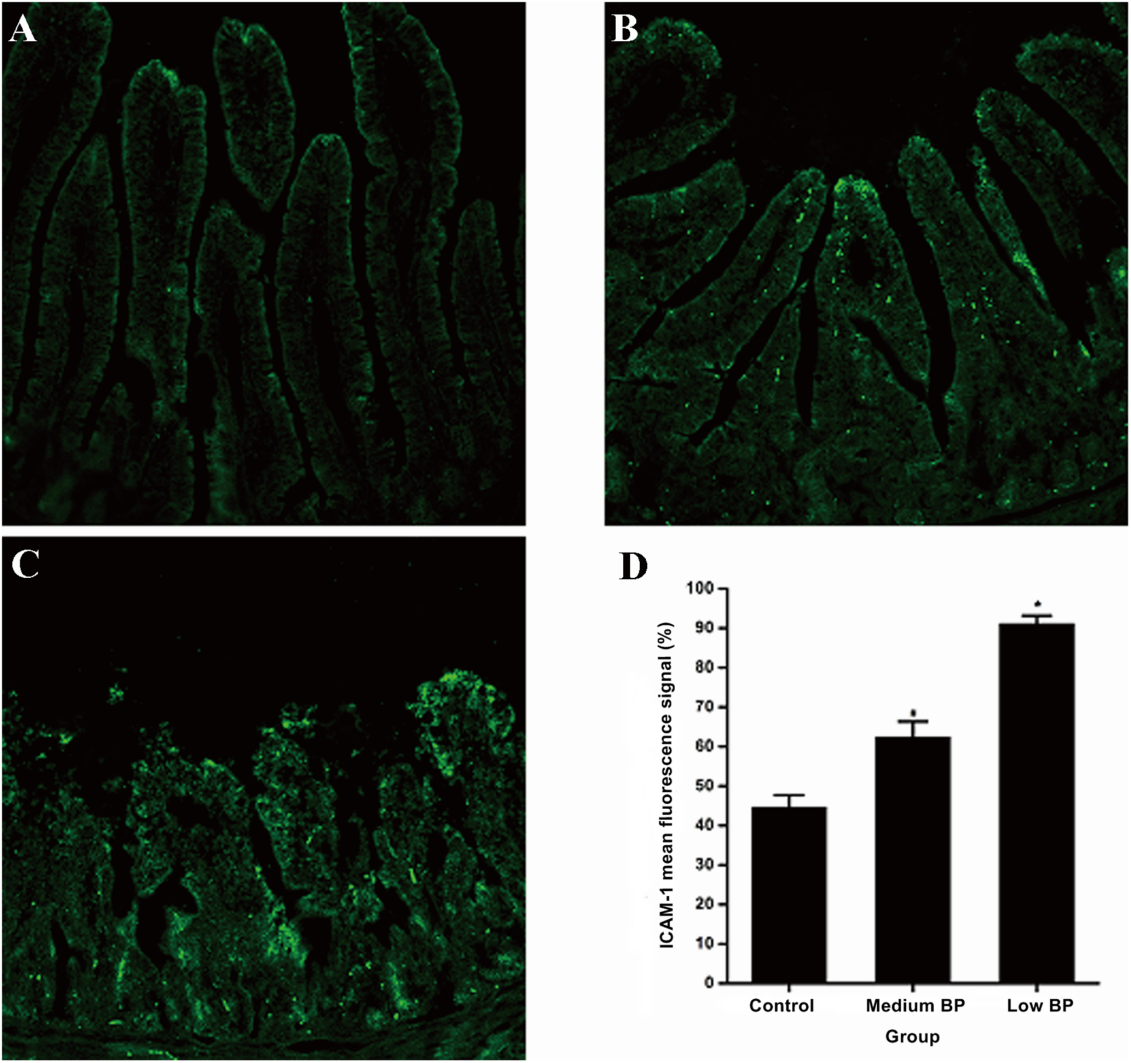
Intestinal ICAM-1 Expression. A: control group; B: medium BP group; C: low BP group; and D: mean fluorescence signal of ICAM-1 (100%). Animals in the medium BP group or low BP group underwent hemorrhagic shock for 180 min at 41–50 mm Hg or 31–40 mm Hg, respectively, before fluid resuscitation. The control group was the sham shock group. Data are shown as means ± SDs. *P<0.05 vs. control group or medium BP group, ^#^ *P*<0.05 vs. control group.

To understand the effects of ICAM-1 on intestinal neutrophil infiltration, ICAM-1 levels were evaluated in our prolonged hemorrhagic shock model (Fig. 4). The minimum fluorescence intensity for ICAM-1 was found in the intestinal mucosa of the control group, while the fluorescence intensity of ICAM-1 staining was significantly increased in the medium and low BP groups (*P*<0.05). Moreover, ICAM-1 fluorescence intensity was significantly higher in the low BP group as compared to the medium BP group (*P*<0.05).

### Intestinal MPO Activity

**Figure 5:**
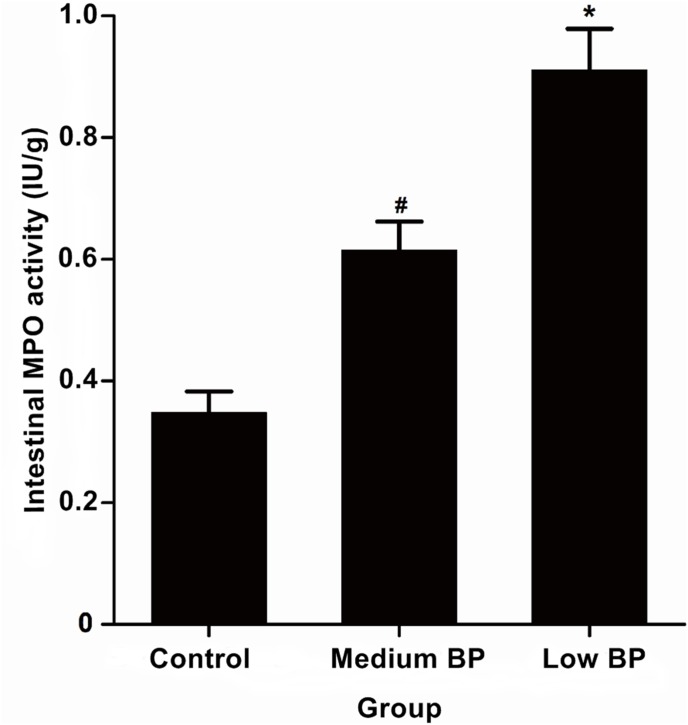
Intestinal Myeloperoxidase (MPO) Activity. Animals in the medium BP group or low BP group underwent hemorrhagic shock for 180 min at 41–50 mm Hg or 31–40 mm Hg, respectively, before fluid resuscitation. The control group was the sham shock group. Data are shown as means ± SDs. *P<0.05 vs. control group or medium BP group, ^#^ *P*<0.05 vs. control group.

As intestinal MPO activity is considered an index of polymorph nuclear leukocyte (PMN) sequestration [34], [35], MPO activity was assessed in our prolonged hemorrhagic shock model (Fig. 5). Compared with the control group (0.348±0.035 IU/g), significantly increased MPO activity was found in the intestines of the medium BP group (0.615±0.047 IU/g, *P*<0.01) and the low BP group (0.911±0.068 IU/g, *P*<0.01). The low BP presented a significantly higher intestinal MPO activity than the medium BP group (*P*<0.01). At T3, statistical analysis showed a negative correlation between the MPO activity and the claudin-1 expression (R = −0.937, *P*<0.001).

### Serum TNF-α

**Figure 6:**
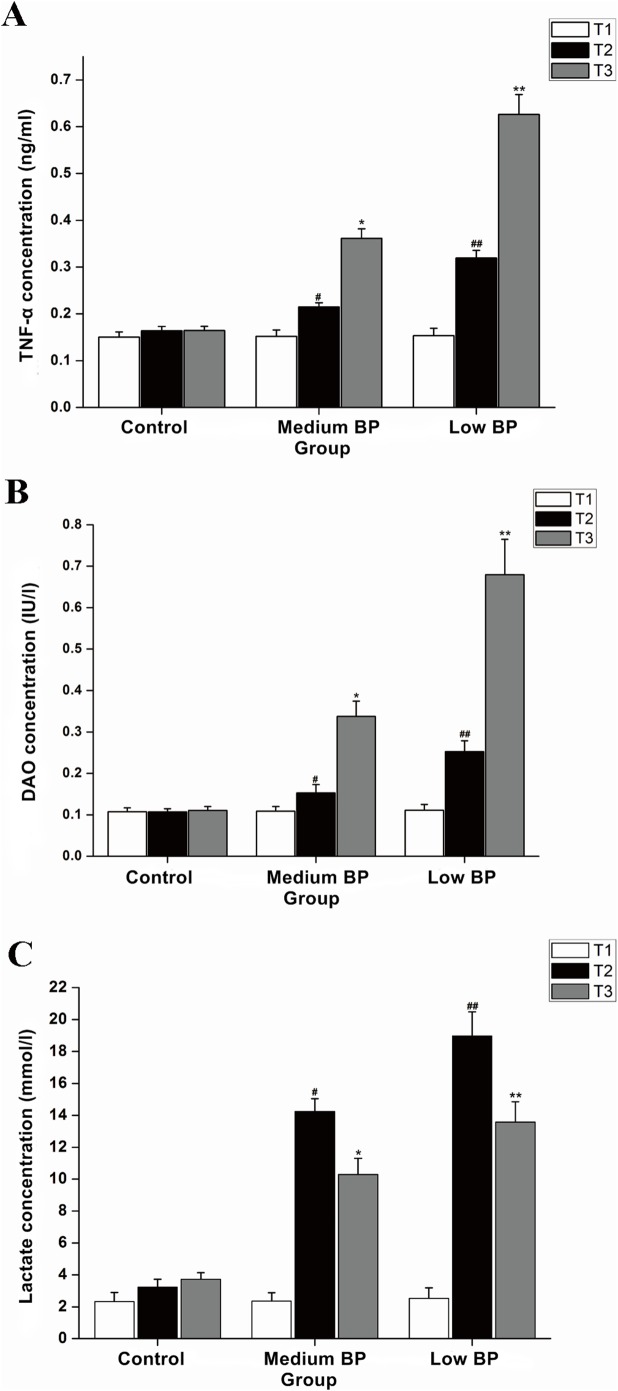
Serum Tumor Necrosis Factor (TNF)-α, Serum Diamine Oxidase (DAO), and Arterial Lactate Levels. A: serum TNF-α; B: serum DAO; and C: arterial lactate. Animals in the medium BP group or low BP group underwent hemorrhagic shock for 180 min at 41–50 mm Hg or 31–40 mm Hg, respectively, before fluid resuscitation. The control group was the sham shock group. Data are shown as means ± SDs. *P<0.05 vs. control group or medium BP group, ^#^ *P*<0.05 vs. control group. **P<0.05 vs. control group or medium BP group, ^##^ *P*<0.05 vs. control group.

To assess systemic inflammation, serum TNF-α was measured in our prolonged hemorrhagic shock model (Fig. 6A). During the experiment, serum TNF-α in the control group showed no significant change (*P*>0.05). At T1, serum TNF-α values were not significantly different between binary comparisons of the control group (0.152±0.013 ng/ml), the medium BP group (0.152±0.014 ng/ml), and the low BP group (0.153±0.016 ng/ml) (*P*>0.05). However, at T2 and T3, serum TNF-α levels were significantly different between binary comparisons of the control group, the medium BP group, and the low BP group (T2: 0.164±0.009 ng/ml, 0.215±0.009 ng/ml, and 0.319±0.016 ng/ml, respectively, *P*<0.01; T3: 0.164±0.009 ng/ml, 0.362±0.020 ng/ml, and 0.626±0.042 ng/ml, *P*<0.01). These results indicate that a more severe hemorrhagic shock induces higher serum TNF-α concentrations in the prolonged hemorrhagic shock model, which indicates more severe systemic inflammation. At T3, statistical analysis showed a negative correlation between serum TNF-α and shock BP (R = −0.833, *P*<0.01) and a negative correlation with tissue claudin-1 expression (R = −0.958, *P*<0.01).

### Serum DAO

To assess whether serum DAO can indicate the severity of intestinal damage, serum DAO was detected in our prolonged hemorrhagic shock model (Fig. 6B). At T1, no significant differences in serum DAO levels were detected between the control group and either the medium BP or low BP group (0.107±0.010 IU/l vs. 0.109±0.011 IU/l or 0.111±0.014 IU/l, respectively, *P*>0.05). At T2, serum DAO levels in the medium BP and low BP groups were both significantly higher than that in the control group (0.153±0.020 IU/l and 0.252±0.026 IU/l vs. 0.107±0.008 IU/l, respectively, *P*<0.01). A similar trend occurred at T3 (0.337±0.037 IU/l and 0.670±0.085 IU/l vs. 0.110±0.009 IU/l, respectively, *P*<0.01). Binary comparisons also revealed that the serum DAO level in the medium BP group were lower than that in the low BP group (0.153±0.020 IU/l vs. 0.252±0.026 IU/l at T2, *P*<0.01; 0.337±0.037 IU/l vs. 0.670±0.085 IU/l, *P*<0.01). At T3, statistical analysis of the serum DAO content showed a positive correlation with serum TNF-α (R = 0.970, *P*<0.01) and a positive correlation with Chiu’s score (R = 0.601, *P*<0.01).

### Arterial Lactate

To assess hypoxia and hypoperfusion, arterial lactate was measured in our prolonged hemorrhagic shock model (Fig. 6C). No significant differences in arterial blood lactate were observed between all three groups at T1. Compared with the control group, anticipated increases in lactate levels occurred in the medium BP and low BP groups at T2 (18.97±1.52 M and 14.25±0.80 mM vs. 3.23±0.49 mM, respectively, *P*<0.01) and at T3 (10.29±1.02 mM and 13.58±1.27 mM vs. 3.73±0.40 mM, respectively, *P*<0.01). At T2, the low BP group manifested significantly higher lactate levels compared with the medium BP group (18.97±1.52 mM vs. 14.25±0.80 mM, *P*<0.05); a similar finding was observed at T3 (10.29±1.02 mM vs. 13.58±1.27 mM). After fluid resuscitation, hypoxia and hypoperfusion were alleviated. As a result, the blood lactate concentration at T3 was lower than that at T2 in the medium BP group (*P*<0.05) and the low BP group (*P*<0.05). In the medium and low BP groups, serum lactate displayed an “increase after shock and decrease after resuscitation” trend.

## Discussion

Traumatic hemorrhage remains the most common cause of death in patients under 45 years of age. Particularly in battlefields and remote areas, hemorrhagic shock patients miss the optimal window for resuscitation, often resulting in MOF and death [1]. Based on the observation that leukocyte activation occurs three to six hours following traumatic hemorrhagic shock [16], we proposed three hours of hemorrhagic shock as an absolute minimum window for prolonged hemorrhagic shock. On this basis, a prolonged hemorrhagic shock animal model was constructed with two different levels of shock BP. We found that hemorrhagic shock could be prolonged to three hours if and only if the shock BP was maintained above 30 mm Hg (data not reported). As expected, under the two different BP conditions (41 to 50 mm Hg and 31 to 40 mm Hg), the lower the hemorrhagic shock BP, the more severe the hemorrhagic shock following fluid resuscitation. Both shock BP and BP after resuscitation in the low BP group were significantly lower than in the medium BP group.

The gut is considered to be the “motor” driving MOF [5]–[7]. As a result, intestinal pathological damage was observed in our prolonged hemorrhagic shock model. Chiu’s score showed that the severity of intestinal mucosal damage in the low BP groups was significantly greater than those in the medium BP or control groups. Tight junction proteins, such as claudin-1, are critical structural proteins for maintaining mucosal barrier function [33], and claudin-1’s aberrant expression directly impairs mucosal barrier function. Here, intestinal claudin-1 expression in the low BP group was significantly lower than that in the medium BP group, which, in turn, was significantly lower than that in the control group. This indicator of impaired intestinal mucosal barrier function is consistent with intestinal mucosa damage. Thus, in the prolonged hemorrhagic shock model, a more severe shock appears to induce a more severe intestinal injury.

Intestinal barrier dysfunction can produce systemic inflammation, and TNF-α has been established as an indicator of systemic inflammation. At T2 and T3, serum TNF-α in the medium and low BP groups were significantly higher compared to the control group. Moreover, serum TNF-α in the low BP group was significantly higher than that in the medium BP group. There was a negative correlation between serum TNF-α and hemorrhagic shock BP. These results indicate that the more severe the hemorrhagic shock, the more pronounced the systemic inflammation. Grotz *et al.* reported increases in serum TNF-α and IL-6 after superior mesenteric occlusion [36]. Tamion *et al.* found that serum levels of TNF-α and IL-6 rose more than before fluid resuscitation in rats following hemorrhagic shock [37]. Here, the intestinal injury score in the low BP group was higher than that in the medium BP group, and the serum TNF-α level in the low BP group was also significantly higher than that in the medium BP group. Statistical analysis revealed that serum TNF-α and claudin-1 expression was negatively correlated at T3. These results indicate that the more serious the mucosal barrier damage, the more severe the systemic inflammation.

Conversely, the systemic inflammatory response can also aggravate mucosal barrier injury. Inflammatory cytokines, such as TNF-α and IL-6, promote intestinal leukocyte adhesion and isolation [38]–[40]. Intestinal MPO activity has been described as an index of intestinal polymorphonuclear leukocyte (PMN) sequestration [34], [35]. Here, the lowest level of MPO activity was shown in the control group, a higher level was observed in the medium BP group, and the highest level was measured in the low BP group. Statistical analysis demonstrated that MPO activity and claudin-1 expression were negatively correlated at T3 and further confirmed that leukocyte infiltration occurred in the intestinal tissue. ICAM-1 is one of the immunoglobulins secreted by activated lymphocytes in ischemic and hypoxic conditions that plays an important role in the adhesion between white blood cells and endothelial cells [41], [42]. Here, the control group demonstrated the lowest level of ICAM-1 expression in the intestinal epithelium, the medium BP group a higher level, and the low BP group the highest level. These results demonstrated that leukocyte infiltration promoted intestinal mucosal barrier damage.

DAO is one of the diamine oxidases catalyzed by deaminases that is primarily expressed in the small intestine (and rarely in the serum under normal circumstances) [43], [44] and is metabolized in the liver [45]. When intestinal injury occurs under conditions of reduced intestinal perfusion, tissue DAO levels decrease [30], and serum DAO levels increase [27]–[29]. Relative to the control group, the serum DAO level increased 3–6 fold in the low BP group and 2–3 fold in medium BP group. Statistical analysis showed a positive correlation between the serum DAO levels and Chiu’s score and a negative correlation between serum DAO levels and intestinal claudin-1 expression. Increased serum DAO levels consistently reflect an elevated degree of intestinal mucosal barrier injury.

As blood lactate typically increases from anaerobic glycolysis due to inadequate oxygen delivery, increased blood lactate is an indicator of metabolic acidosis. It is widely accepted that lactate is a clinical indicator of the degree of traumatic hemorrhagic shock [46]. Physiologically, increases in lactate levels typically demonstrate tissue hypoperfusion and/or increases in anaerobic metabolism [47]. Our results showed that, after hemorrhagic shock and reperfusion, lactate levels were still significantly higher than normal, but there was a notable decrease in lactate levels. The reasons for this decline in lactate levels are speculated to be: (i) upon fluid resuscitation, blood perfusion improves, which in turn, improves oxygen delivery and aerobic metabolism, thereby lowering circulating lactate levels; and (ii) upon fluid resuscitation, blood volume increases, thereby lowering lactic acid levels by dilution. That being said, these changes in lactate levels did not correlate with the organ damage after prolonged hemorrhagic shock resuscitation. However, statistical analysis did indicate the change in serum DAO was correlated with the change in TNF-α, which reveals serum DAO may be a potential indicator of the severity of prolonged hemorrhagic shock.

### Limitations

Although our experiments confirmed that serum DAO levels reflect the severity of intestinal injury and elevated serum DAO levels are associated with increased degrees of systemic inflammation in a rabbit model, serum DAO’s role as a potential indicator of the severity of prolonged hemorrhagic shock requires further study in clinical trials.

## Conclusions

In this study, we constructed a prolonged hemorrhagic shock animal model demonstrating that a more pronounced shock BP induces increased levels of systemic inflammation and intestinal mucosal barrier damage, suggesting that systemic inflammation possesses an etiopathological relationship with intestinal injury. The results reveal that serum DAO can reflect the severity of intestinal damage in prolonged hemorrhagic shock. Moreover, statistical analyses reveals serum DAO shows potential in evaluating the severity of prolonged hemorrhagic shock.
